# Construction of an Expression Vector Containing Mtb72F of *Mycobacterium tuberculosis*

**Published:** 2012-06-13

**Authors:** Maryam Sadat Nabavinia, Mahboobeh Naderi Nasab, Zahra Meshkat, Mohammad Derakhshan, Mehrangiz Khaje-Karamadini

**Affiliations:** 1. Microbiology and Virology Research Center, Mashhad University of Medical Sciences ,Mashhad,Iran; 2. Department of Medical Bacteriology and Virology, Emam Reza Hospital, Faculty of Medicine, Mashhad University of Medical Sciences, Mashhad, Iran; 3. Women's Health Research Center, Mashhad University of Medical Sciences, Mashhad, Iran; 4. Department of Medical Bacteriology and Virology, Qaem Hospital, Faculty of Medicine, Mashhad University of Medical Sciences, Mashhad, Iran

**Keywords:** *Mycobacterium tuberculosis*, Vaccine, Mtb72F

## Abstract

**Objective:**

Despite using the Bacille Calmette Guerin (BCG) vaccine, tuberculosis (TB) is still a worldwide disease that kills 2-3 million people each year. Developing a new and more effective vaccine is one way to possibly reduce the morbidity and mortality of TB. The Mtb72F vaccine is one of the important subunit vaccines applied in human clinical trials. In this study, we have constructed an expression vector that contains the Mtb72F fragment with some new modifications.

**Materials and Methods:**

In this experimental study, Mtb32N and Mtb39 fragments were amplified by polymerase chain reaction (PCR) using specific primers and inserted into pET21b\Mtb32C. Colony-PCR, restriction enzyme analysis, and DNA sequencing were used to confirm the accuracy of the cloning. We used Western blot to verify the desired protein expression.

**Results:**

The amplified fragments showed the desired size in PCR and digestion methods, and protein expression was confirmed using a monoclonal antibody.

**Conclusion:**

Our modification made it possible to insert another gene or gene fragments into the Mtb72F vector for developing new constructs. In addition, our data has shown that the placement of the histidine tag in the carboxyl- (C-) or amino- (N-) terminal part of a protein may influence protein expression and/or stability.

## Introduction

According to the World Health Organization (WHO), about 9 million people are affected by progressive tuberculosis (TB) each year. As *Mycobacterium tuberculosis* (*M. tuberculosis*) can induce active disease in 10% of infected individuals, it is estimated that one-third of the world's population is infected (1). The spreading of multi-drug resistance (MDR) *M. tuberculosis* also makes it a serious threat for world health (2). Development of an effective vaccine against TB is the only hopeful way to control this threatening disease. The Bacille Calmette Guerin (BCG) vaccine has been widely used to prevent TB since the 1950’s. Nowadays, studies have shown that the BCG vaccine is useful for the prevention of meningitis in in neonates, children and military TB, but it is not effective for pulmonary TB in adults. In addition, the BCG vaccine is not protective against established latent infections (3). Several studies have shown that the BCG vaccine is not proper for use in human immunodeficiency virus (HIV)-infected individuals or for administration as a booster vaccine in adults. The vaccine is also not effective in tropical regions (4, 5). Therefore an investigation on new vaccines has continued in order to develop an effective and safe vaccine, especially for immunocompromised individuals. New vaccines should generate cell-mediated immunity responses, including Th1, T CD8+, and CD1 restricted αβ T-cells. IFNλ production by T-cells is another important aspect in designing a new vaccine (6). Several studies have tried to locate new immunogenic antigens of *M. tuberculosis* which can be used for the construction of new effective vaccines that would be safe in vaccinated subjects. About 200 vaccines have been developed against TB; however only eight, including MTB72F, were effective in the animal model (6, 7). MTB72F, a fusion protein vaccine, was constructed by linking the Mtb32 (RV0125) and Mtb39 (RV1996) genes of *M. tuberculosis* (H37Rv strain) (8). Mtb32, a serine protease protein, was discovered by the culture filtrate protein technique. *in vitro*, this protein has been shown to induce T-cell proliferation and IFNλ production in peripheral blood mononuclear cells in a purified protein derivative (PPD) skin test-positive healthy individual (9). Mtb39A is a member of the PPE family in *M. tuberculosis*, which causes T-cell proliferation and IFNλ production in vaccinated subjects. The induction of maximum responses in very low concentrations of protein (10 ng/ml) is an important aspect of the Mtb39A protein.

Previous studies have shown that Mtb72F is able to induce more efficient immune responses in vaccinated animals than either Mtb32 or Mtb39 alone (10). The vaccine is presently under phase II of human clinical trials (11). Other studies have shown that administration of Mtb72F as a booster vaccine after the BCG vaccine protects different animals, such as guinea pigs and cynomolgus monkeys, against *M. tuberculosis*. The vaccine has also been determined to be safe in the monkey model (12, 13). Similar to BCG, the Mtb72F vaccine can protect vaccinated rabbits against TB meningitis (14). The aim of this study is the construction of the Mtb72F expression vector by fusing the Mtb39 between the C- and N- terminal of the Mtb32 gene of *M. tuberculosis*. The desired vector could be used for preparation of the Mtb72F protein as a potent protein vaccine.

## Materials and Methods

### Cloning of Mtb32N into pET21b/Mtb32C

 The pET21b/Mtb32C vector was constructed as previously described (15). In this study, the Mtb32N fragment and the Mtb39 gene were inserted into the previous construct. Given the sequence of the Mtb32N fragment (H37Rv strain, GenBank: BX842572.1) and Mtb39 gene (H37Rv strain, GenBank: BX842575.1) of *M. tuberculosis*, the two pairs of PCR primers were designed to amplify the desired target regions. For evaluating the specificity of the designed primers, we tested the primers on different registered sequences in GenBank using the NCBI Blast Resource. Since the full lengths of the genes were needed, designing the primers from various locations was not possible and the PCR set up was only performed by changing the PCR program and mixture to obtain the desired fragments. In addition, the amplified fragments were cloned and sequenced for confirmation of the insertion of desired fragments in correct locations and suitable orientations. First, the Mtb32N fragment was amplified by PCR from the genomic DNA of *M. tuberculosis* using the specific primers: 5´-CTAATC**GAATTC**GCCCCGCCGGCCTTGTCGCAGGAC-3´ as a forward primer and 5´-TAATC**AAGCTT**CTATCAgtgatggtgatggtgatgGGACGCGGCCGTGT-3´ as a reverse primer (H37Rv strain, GenBank: BX842572.1). The underlined letters represent the *Eco*RI restriction enzyme site on the forward primer and the *Hind*III restriction enzyme site on the reverse primer. Italic and bold letters represent two stop codons on the reverse primer, which are followed by 18 small letters that encode for 6 histidine amino acids. The PCR mixture consisted of 0.3 µl of 10 pmol primers, 0.3 µl dNTP (CinnaGen, Iran), 2.5 unit *Pfu* enzyme (Fermentas, Korea), 1.5 µl *Pfu* buffer with MgSO4, and 1 µl DNA (150 ng/µl) for a total volume of 15 µl.

 The Mtb32N gene was amplified by touchdown PCR using the following program: initial denaturation at 95℃ for 10 minutes, 25 cycles that (the temperature was decreased 0.5℃ in each cycle), and 2 minutes at 72℃; followed by 12 cycles with 1 minute at 95℃, 50 seconds at 59.5℃, and 2 minutes at 72℃. The final extension was performed at 72℃ for 10 minutes.

The PCR product of the amplified Mtb32N fragment (about 150 µl) was used in an agarose gel electrophoresis for future purification. The specific band was extracted from the agarose gel using a commercial kit (Bioneer, Korea) according the manufacturer's recommendations, and then digested by *Eco*RI and *Hind*III restriction enzymes (16). The vector, pET21b\Mtb32C, was purified using a mini prep kit based on the manufacturer's instructions (Fermentas Company), digested by *Eco*RI and *Hind*III restriction enzymes, and purified from an agarose gel as described above. The DNA *T4 ligase* enzyme was used to insert the digested and purified Mtb32N fragment into the digested vector. The ligation mixture consisted of 1 µl Mtb32N fragment (60 ng/µl), 3 µl pET21b/Mtb32N (60 ng/µl), 2 unit *T4 DNA ligase* enzyme (Fermentas Company), 2 µl *T4 DNA ligase* buffer, and 6 µl of DNase-free water for a total volume of 20 µl. The prepared mixture was incubated overnight at 22℃. Competent *E. coli* DH5α was prepared using 50 mM cold CaCl_2_ (17) and transformation of the competent bacteria was done using the heat shock method (90 seconds at 42℃) (16). The transformed bacteria were cultured on LB agar that contained 100 µg/ml ampicillin and incubated at 37℃ for about 16 hours (18). The presence of the Mtb32N was confirmed by colony-PCR using specific primers and restriction enzyme analysis.

### Cloning of Mtb39 into pET21b/Mtb32C/Mtb32N for the construction of Mtb72F

PCR was performed for the amplification of Mtb39 from genomic DNA of the bacteria (H37Rv strain, GenBank: BX842575.1). Two specific primers, 5´-CTAATCGGATTCATGGTGGATTTCGGGGCGTTA-3´ as a forward primer and 5´-CTAATC**GAATTC**GCCGGCTGCCGGAGAATGCGG-3´ as a reverse primer were used for PCR amplification (underlined letters in the forward primer show the *Bam*HI enzyme restriction site and in the reverse primer show the *Eco*RI enzyme restriction site). The PCR mixture was prepared as described above and the PCR program consisted of three steps: the first step consisted of denaturation at 95℃ for 10 minutes; the second step consisted of 35 cycles at 95ºC for 1 minute, 54ºC for 1 minute and 72ºC for 2.5 minutes; and the third step was the final extension at 72ºC for 10 minutes. Amplified fragments and the vector were digested using *Bam*HI and *Eco*RI restriction enzymes and purified as described above. The ligation step and transformation of the competent bacteria were similar to Mtb32N, as described above. The final recombinant vector, Mtb72F, was confirmed by colony-PCR, restriction enzyme analysis, and sequencing (Milligene Company, France) (16).

### Sodium dodecyl sulfate-polyacryl amide gel electrophoresis (SDS-PAGE) and Western blot

For protein expression, *E. coli* BL21b was cultured in 2*YT medium and the competent cells were prepared using cold CaCl_2_ as described in previous sections. The Mtb72F vector was propagated and purified from *E. coli* DH5α and then transformed into competent *E. coli* BL21b using the heat shock method. The transformed bacteria were cultured in 2*YT medium that contained 50 µg/ml ampicillin. For protein expression, 2 µM isopropyl-β-D-thio-galactoside (IPTG) was used for bacteria induction (OD: 0.5 at 600 nm). Four hours after induction, the bacteria were mixed with electrophoresis sample buffers and utilized in SDS-PAGE and Western blot using a monoclonal antibody (mouse anti-histidine Tag; AbD Serotec, UK) (16).

## Results

**Fig 1:**
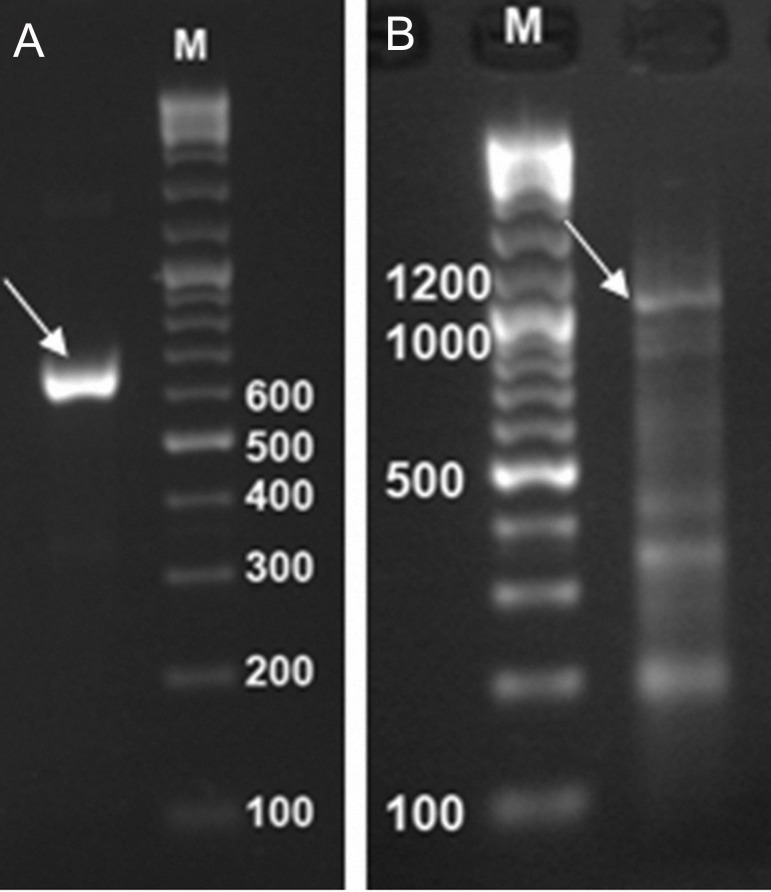
PCR products of the amplified fragments. Mtb32N fragment 632bp (A), Mtb39 fragment 1197bp (B), and 100 bp DNA size marker (M).

As described in the previous section, Mtb32C, Mtb39, and Mtb32N were amplified by PCR using specific primers. The PCR amplified fragments showed the expected size of 632 bp for the Mtb32N fragment and 1197 bp for the Mtb39 fragment (Fig 1).

**Fig 2:**
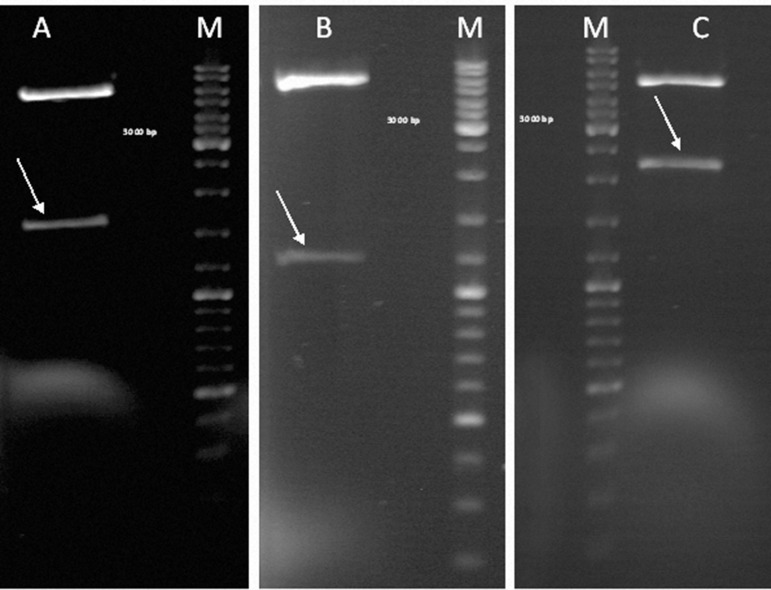
Restriction enzyme analysis of the final construct using different enzymes: Lane A (arrow): double digestion using NdeI and EcoRI enzymes (1600 bp fragment consisted of Mtb32C and Mtb39); Lane B (arrow): double digestion using BamHI and EcoRI enzymes (1200 bp fragment of Mtb39); Lane C (arrow): double digestion using NdeI and HindIII enzymes (2200 bp fragment consisted of Mtb32C, Mtb39, and Mtb32N).

Colony-PCR was performed to confirm the correctness of the cloning process and the existence of the desired fragments in our construct. Positive colonies were selected, propagated, purified, and used for restriction enzyme analysis. Several restriction enzymes were used for digestion. Restriction enzyme analysis of the final construct showed a 1600 bp fragment that used *Nde*I and *Eco*RI enzymes which corresponded to the Mtb32C and Mtb39 fused fragments, a 1200 bp fragment of Mtb39 that used *Bam*HI and *Eco*RI enzymes, and a 2200 bp fragment that consisted of the final fusion construct which included Mtb32C, Mtb39, and Mtb32N fragments that used *Nde*I and *Hind*III enzymes (Fig 2).

The final construct was confirmed by sequencing, and its results compared to other genes already in GenBank (using NCBI Nucleotide BLAST). The sequencing results showed that mutations or deletions did not occur during gene amplification or cloning. In addition, the sequencing of cloned fragments showed >99% identity and a 0% gap compared to the registered Mtb32 and Mtb39 gene sequence of the *M. tuberculosis* H37Rv strain.

**Fig 3:**
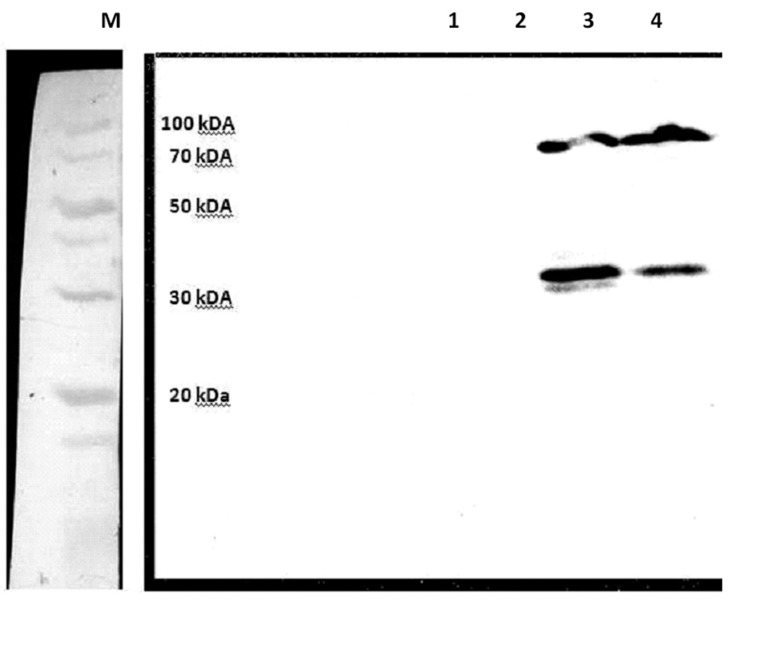
Western blot results to confirm protein expression. Lanes 1 and 2: E. coli BL21 containing an empty pET21 vector. Lanes 3 and 4: E. coli BL21 consisting of the Mtb72F protein.

According to Western blot, there were two protein bands (about 35 and 72 KDa) in which the 35 KDa fragment showed degradation of the recombinant protein after its induction (Fig 3).

## Discussion

BCG is a live attenuated mycobacterial strain that has been used as a preventive vaccine since 1921 (11). Although the BCG vaccine is in wide use worldwide, its protection is not complete and long lasting; thus TB remains a public health problem with about 2–3 million deaths per year (13, 19). Therefore, the development of a new effective vaccine against TB is an important aim for controlling this contagious infection. In this regard, several strategies and/or new vaccines, such as repeat immunizations with BCG, recombinant BCG vaccine, DNA vaccines, subunit vaccines, and fusion protein-based vaccines have been developed. These vaccines have been designed to replace the BCG vaccine or to improve the immune response in individuals previously vaccinated with BCG. However, only a few have shown some success in the first phase of clinical testing (7, 13, 20, 21).

The Mtb72F subunit vaccine is the only TB vaccine candidate that has been effective in four animal models (13, 22, 23). In addition, it is the first TB recombinant vaccine currently in a phase I human clinical trial (8). Therefore additional investigation is necessary to determine its safety and efficacy among different populations.

TB is a major health problem in the world, especially in developing countries. The reported incidence rate in Iran has varied in different provinces, with the highest rate in Sistan and Baloochestan (43.83/100000) (24). With the increasing prevalence of TB in Iran, there is a need to develop new vaccines in order to enhance immune responses in BCG vaccinated individuals and to prevent the increasing incidence and prevalence rates of TB.

With this goal, the construction of the Mtb72F vector has been performed as described by Skeiky et al. (8), with some modifications. In our construct, the Mtb32N fragment was inserted using two restriction enzymes (*Eco*RI and *Hind*III), thus making it possible to insert another fragment in the C-terminal of the fragment for future studies. This modification reduced several steps during the study, such as determining the orientation of the cloned fragment. In addition, a histidine tag was linked to the carboxyl terminal of the new recombinant protein; its expression confirmed the accuracy of the full-length protein translation. For simplification of the expression, the final construct was transformed into *E. coli* BL21 (DE3) without the pLasE plasmid, which also eliminates several practical steps. Although intact Mtb32 fragments show protease activity, separation of the C-terminal from the gene and cloning the fragment upstream of the other gene, (Mtb39 in our study) inactivates the Mtb32 protease activity. A pLas plasmid is not required because plasS and PlasE plasmids are used to express toxic proteins.

Previous studies have shown that the linkage of the NusA protein in the N-terminal of recombinant proteins has led to an increase in their stability in pET systems (25). In addition, other studies have shown that extra N-terminal methionine residues affected protein stability (26). Another important aspect of the present study was degradation of the recombinant protein. In our study, the position of the histidine tag and the expressing host were different from the Skeiky et al. study (8). Therefore, insertion of the histidine tag in the C-terminal of a protein and application of the different expressing hosts may lead to an alteration of protein stability. More studies are needed to evaluate this.

*M. tuberculosis* has been divided into three groups based on the polymorphisms of catalase peroxidase and the A subunit of the Gyrase gene (27). In a study, Sreevatsan et al. detected some polymorphisms in Rv1196 and Rv0125; they have stated that the MTB72F vaccine might not be effective in some populations (28). Therefore, it is necessary to determine the sequence of genes in each population to develop new TB vaccines and to evaluate their distribution in each country so we can use the predominant genotype to design TB vaccines. Construction of a new vaccine containing the Mtb72F from another *M. tuberculosis* genotype will be simple according to the methods that have been used in our study.

MTB72F could serve as a basis for further studies on the development of a new subunit and DNA vaccines against TB. In addition, the MTB72F construct can be used in association with other *M. tuberculosis* antigens to improve the efficacy of the vaccine for TB prevention or treatment.

## Conclusion

The modified Mtb72F construct is suitable for inserting another fragment in order to develop new vaccines. In addition, the production of two separate protein bands in Western blot may indicate that the place of the histidine tag in the C- or N-terminal part of the protein can influence protein expression and/or stability.

## References

[1] World Health Organization (2010). Global Tuberculosis Control Epidemiology, Strategy, Financing. WHO report 2009.

[2] Raviglione MC, Snider DE Jr, Kochi A (1995). Global epidemiology of tuberculosis. Morbidity and mortality of a worldwide epidemic. JAMA.

[3] Colditz GA, Brewer TF, Berkey CS, Wilson ME, Burdick E, Fineberg HV (1994). Efficacy of BCG vaccine in the prevention of tuberculosis. Meta-analysis of the published literature. JAMA.

[4] O'Brien KL, Ruff AJ, Louis MA, Desormeaux J, Joseph DJ, Mc Brien M (1995). Bacillus Calmette-Guérin complications in children born to HIV-1-infected women with a review of the literature. Pediatrics.

[5] Andersen P, Doherty TM (2005). The success and failure of BCG implications for a novel tuberculosis vaccine. Nat Rev Microbiol.

[6] Hoft DF (2008). Tuberculosis vaccine development: goals, immunological design, and evaluation. Lancet.

[7] Singhal N, Bisht D, Joshi B (2010). Immunoprophylaxis of tuberculosis: an update of emerging trends. Arch Immunol Ther Exp (Warsz).

[8] Skeiky YA, Alderson MR, Ovendale PJ, Guderian JA, Brandt L, Dillon DC (2004). Differential immune responses and protective efficacy induced by components of a tuberculosis polyprotein vaccine, Mtb72F, delivered as naked DNA or recombinant protein. J Immunol.

[9] Skeiky YA, Lodes MJ, Guderian JA, Mohamath R, Bement T, Alderson MR (1999). Cloning, expression, and immunological evaluation of two putative secreted serine protease antigens of Mycobacterium tuberculosis. Infect Immun.

[10] Dillon DC, Alderson MR, Day CH, Lewinsohn DM, Coler R, Bement T (1999). Molecular characterization and human T-cell responses to a member of a novel Mycobacterium tuberculosis mtb39 gene family. Infect Immun.

[11] McShane H (2009). Vaccine strategies against tuberculosis. Swiss Med Wkly.

[12] Brandt L, Skeiky YA, Alderson MR, Lobet Y, Dalemans W, Turner OC (2004). The protective effect of the Mycobacterium bovis BCG vaccine is increased by coadministration with the Mycobacterium tuberculosis 72-kilodalton fusion polyprotein Mtb72F in M. tuberculosis-infected guinea pigs. Infect Immun.

[13] Reed SG, Coler RN, Dalemans W, Tan EV, DeLa Cruz EC, Basaraba RJ (2009). Defined tuberculosis vaccine, Mtb72F/AS02A, evidence of protection in cynomolgus monkeys. Proc Natl Acad Sci U S A.

[14] Tsenova L, Harbacheuski R, Moreira AL, Ellison E, Dalemans W, Alderson MR (2006). Evaluation of the Mtb72F polyprotein vaccine in a rabbit model of tuberculous meningitis. Infect Immun.

[15] Nabavinia MS, Naderi-Nasab M, Meshkat Z, Derakhshan M, Khajeh-Karamadini M (2011). Construction and evaluation of an expression vector containing Mtb32C (Rv0125) of Mycobacterium tuberculosis. Avicenna J Med Biotech.

[16] Nabavinia MS (2011). Construction of Mtb72F expression vector by fusing the Mtb39 and Mtb32 genes of Mycobacterium tuberculosis. Presented for the M.Sc..

[17] Sambrook J, Russell DW (2001). Molecular cloning: a laboratory manual.

[18] Meshkat Z (2007). Construction of an expression vector containing immunogenic region of human papillomavirus type 16 E7 and HSP70 genes and evaluate the CMI responses in BALB/c mice. Presented for the Ph.D..

[19] Dye C, Raviglione M (2005). Monitoring global health: WHO has mandate and expertise. BMJ.

[20] Griffin JF, Mackintosh CG, Rodgers CR (2006). Factors influencing the protective efficacy of a BCG homologous prime-boost vaccination regime against tuberculosis. Vaccine.

[21] McShane H, Hill A (2005). Prime-boost immunisation strategies for tuberculosis. Microbes Infect.

[22] Reed SG, Alderson MR, Dalemans W, Lobet Y, Skeiky YA (2003). Prospects for a better vaccine against tuberculosis. Tuberculosis (Edinb).

[23] Reed S, Lobet Y (2005). Tuberculosis vaccine development; from mouse to man. Microbes Infect.

[24] Ebrahimzadeh A, Sharifzadeh GHR, Eshaghi S (2009). The epidemiology of Tuberculosis in Birjand (1996-2006). JBUMS.

[25] De Marco V, Stier G, Blandin S, de Marco A (2004). The solubility and stability of recombinant proteins are increased by their fusion to NusA. Biochem Biophys Res Commun.

[26] Uversky VN, Abdullaev ZK, Arseniev AS, Bocharov EV, Dolgikh DA, Latypov RF (1999). Structure and stability of recombinant protein depend on the extra N-terminal methionine residue: S6 permutein from direct and fusion expression systems. Biochim Biophys Acta.

[27] Sreevatsan S, Pan X, Stockbauer KE, Connell ND, Kreiswirth BN, Whittam TS (1997). Restricted structural gene polymorphism in the Mycobacterium tuberculosis complex indicates evolutionarily recent global dissemination. Proc Natl Acad Sci USA.

[28] Hebert AM, Talarico S, Yang D, Durmaz R, Marrs CF, Zhang L (2007). DNA polymorphisms in the pepA and PPE18 genes among clinical strains of Mycobacterium tuberculosis: implications for vaccine efficacy. Infect Immun.

